# Recurrent Infections in Allergic Pediatric Patients: An Immune System Problem? A Narrative Review

**DOI:** 10.3390/children12060788

**Published:** 2025-06-17

**Authors:** César Galván, Rafael Durán, Cristian Matos, Cristiana Indolfi, Angela Klain

**Affiliations:** 1 Emedic Salud, Lima 15036, Peru; 2 Human Nutrition and Food Research Group (GINAH), Universidad Científica del Sur, Lima 150142, Peru; 3 Faculty of Medicine, Universidad Peruana Cayetano Heredia, Lima 15102, Peru; cristian.matos@upch.pe; 4 Department of Woman, Child and General and Specialized Surgery, University of Campania ‘Luigi Vanvitelli’, 80138 Naples, Italyangela.klain@studenti.unicampania.it (A.K.)

**Keywords:** pediatric allergies, recurrent infections, immune dysfunction, allergic rhinitis, asthma, innate immunity, adaptive immunity

## Abstract

**Background/Objectives:** Recurring infections in children with allergies pose significant clinical challenges, with these conditions often exacerbating each other through complex immunological interactions. This narrative review examines the connection between recurring infections and allergic conditions in pediatric patients, focusing on how immune system dysfunction influences infection susceptibility in respiratory allergies. **Methods:** A comprehensive literature search across PubMed, Web of Science, and SciELO databases was conducted from January 2014 to May 2024. Studies involving children and adolescents up to 18 years old with diagnosed respiratory allergies were included, while reviews, opinion pieces, case reports, and studies not addressing immune–infection interactions were excluded. **Results:** Analysis reveals significant immune dysfunction in allergic children, affecting both innate and adaptive immunity components. Children with allergic rhinitis and asthma demonstrate decreased interferon-gamma production, increasing vulnerability to viral infections (particularly rhinovirus) and bacterial infections such as Mycoplasma pneumoniae. Rhinovirus represents the most common pathogen, present in 75% of asthma exacerbations. Atopic children exhibit markedly higher bacterial infection rates, with 27.1% showing Mycoplasma pneumoniae involvement versus 4.9% in non-atopic children. **Conclusions:** Recurring infections in allergic pediatric patients result from significant immune dysfunction involving altered cytokine production and immune cell function. These complex interactions highlight the need for targeted therapeutic approaches that enhance immune responses and reduce infection risks. Future research should focus on identifying specific biomarkers and immune mechanisms for developing more effective interventions.

## 1. Introduction

Allergic diseases represent a major global health burden, affecting an estimated 10–20% of children worldwide and contributing substantially to pediatric morbidity and healthcare utilization. Among atopic disorders, allergic rhinitis (AR) and asthma are the most prevalent, with AR affecting up to 40% of school-age children [1] and asthma being present in approximately 10% of this population [2]. These conditions not only impair quality of life but also predispose children to recurrent infections, creating a cyclical interplay between allergies and infection susceptibility.

Recurring infections in children with allergies poses a notable challenge for clinicians and researchers alike. When respiratory allergies and heightened vulnerability to infections occur together, they often worsen each other’s effects. Children affected by this combination may experience both flare-ups of their allergic condition triggered by pathogens and suffer from repeated infectious episodes [3]. Although this clinical overlap is well documented, the underlying immunological mechanisms remain incompletely understood. The underlying immune response disruptions vary considerably among patients: some show elevated levels of inflammatory cytokines [4,5] while others display marked reductions in regulatory T cell populations [6]. Respiratory allergic conditions like AR tend to trigger intensified immune reactions against everyday allergens, creating persistent inflammation throughout the body [7], potentially setting the stage for infections to take hold.

Viral pathogens, particularly human rhinoviruses (HRVs), respiratory syncytial virus (RSV), and influenza viruses, are the leading triggers of these recurrent respiratory infections in allergic children, with HRV detected in up to 75% of asthma exacerbations [8]. Antiviral defense typically begins with pathogen recognition via pattern recognition receptors such as Toll-like receptors (TLR3, TLR7) and RIG-I–like receptors. These receptors initiate downstream signaling cascades involving IRF3 and IRF7 transcription factors, which subsequently drive the production of type I (IFN-α/β) and type III (IFN-λ) interferons. These interferons then activate a wide array of interferon-stimulated genes (ISGs), promoting viral clearance by limiting viral replication and enhancing immune cell activation [9,10]. In allergic individuals, however, a Th2-skewed immune environment—characterized by elevated IL-4, IL-5, and IL-13—can suppress this antiviral pathway. These cytokines have been shown to inhibit interferon production, impair dendritic cell and natural killer (NK) cell function, and compromise epithelial barrier integrity, ultimately weakening the innate antiviral response [9,10,11].

As a result of this immune dysfunction, any malfunction in the immune system’s protective mechanisms can leave allergic children more susceptible to infectious diseases. Research has found that children suffering from AR often produce insufficient amounts of interferon-gamma (IFN-γ), a key antiviral cytokine, potentially explaining their higher rates of infection [12,13].

The interplay between allergic conditions and infections takes on particular importance in children, who appear especially vulnerable to both health issues. Viral respiratory infections frequently affect children with allergies and asthma, with rhinovirus (RV) emerging as one of the most common culprits [14,15]. This suggests a two-way relationship where allergic disorders may increase infection susceptibility, and these infections may subsequently intensify allergic symptoms. Additionally, bacterial pathogens like Mycoplasma pneumoniae (Mp) and Streptococcus pneumoniae have been associated with more intense asthma flare-ups, highlighting why it is needed to better understand these complex interactions [16,17,18].

This review aims to synthesize the current evidence on immune dysfunction in allergic pediatric patients and its role in recurrent infections. It focuses particularly on the mechanisms of innate and adaptive immunity, including antiviral cytokine responses and Th2-skewed immune profiles, to explore how immune deviation in allergic children contributes to infectious susceptibility. By clarifying what is currently understood and highlighting knowledge gaps, this review seeks to guide clinicians in improving management strategies and to support researchers in identifying future investigative priorities.

## 2. Literature Search

This was a narrative review that incorporated some systematic search elements to comprehensively capture the relevant literature on the relationship between recurrent infections and allergies in pediatric patients, specifically focusing on whether frequent infections are a result of a weakened immune system and whether exposure to certain pathogens can exacerbate allergies. First, a systematic literature search was conducted in PubMed, Web of Science, and SciELO, targeting the literature published between January 2014 and May 2024. The search in PubMed was run using the following MeSH terms: (“Respiratory Hypersensitivity” OR “Allergy and Immunology” OR “Rhinitis, Allergic” OR “Asthma”) AND (“Bacterial Infections and Mycoses” OR “Virus Diseases”) AND (“Immunologic Deficiency Syndromes” OR “Immune System Diseases”) AND (“Child” OR “Adolescent”). The search in Web of Science employed similar terms: (((Allergic rhinitis OR Allergic asthma OR Respiratory allergic diseases) AND (Bacterial Infections and Mycoses OR Virus Diseases)) AND (Immunologic Deficiency Syndromes OR Immune System Diseases)) AND (child OR Adolescent). For SciELO, the search was conducted with terms translated into Spanish: (((((((Hipersensibilidad Respiratoria) OR (Alergia e Inmunología)) OR (Rinitis Alérgica)) OR (Asma)) AND (Infecciones Bacterianas y Micosis OR Enfermedades por Virus)) AND (Síndromes de Inmunodeficiencia OR Enfermedades del Sistema Inmunológico)) AND (Niño)). The initial searches yielded a total of 1034 articles from PubMed, 18 articles from Web of Science, and 19 articles from SciELO. Second, a manual search was conducted through Google Scholar, which added 8 relevant articles to the review.

## 3. Study Eligibility, Selection, and Data Extraction

The search focused on studies involving children and adolescents up to 18 years old diagnosed with respiratory allergies. The objective was to evaluate the relationship between recurrent infections and the immune system in this patient group. In total, 1059 articles were initially identified across all databases. After removing duplicates and applying the predefined exclusion criteria, 46 articles were ultimately included. Additionally, 11 papers were manually added to the final selection, resulting in a total of 57 articles included in the review, while 1013 were excluded. The exclusion criteria were as follows: non-original articles such as reviews, editorials, and case reports; articles that did not focus exclusively on pediatric populations; and studies that lacked specific discussion of immune response and infection in the context of respiratory allergies.

The screening process was conducted using the Rayyan platform, and all articles were exported to the reference manager EndNote 20. Initially, a search was performed to identify potential duplicates, followed by a review of titles and abstracts to carry out the first screening. Subsequently, a second analysis was conducted to assign labels to the articles, focusing on disease exacerbations, the type of disease or pathogen, and the affected immunity mechanism.

## 4. Affected Immunity Mechanism

Allergies are conditions directly related to the immune system, originating from excessive immune reactions to normally harmless substances. In susceptible individuals, these substances can present allergenic potential, triggering the production of specific antibodies by the immune system [9].

The immune system has two main categories of defense: innate immunity and adaptive immunity. Innate immunity consists of physical and chemical barriers as the body’s first line of defense against infections, as well as cells and proteins specifically designed to recognize pathogens. Among these cellular agents are macrophages and neutrophils, which aim to phagocytize and destroy invading agents. Additionally, the complement system, composed of an enzymatic cascade and cytokines, functions to amplify the inflammatory response and recruit more immune cells [9,19].

The adaptive immune response, which typically becomes activated after the innate system has responded, targets pathogens with greater precision and falls into two main categories: humoral immunity and cell-mediated immunity (CMI). B lymphocytes drive humoral immunity by generating pathogen-specific antibodies, while T lymphocytes form the backbone of CMI, eliminating infected cells and amplifying immune reactions through cytokine secretion [9,12]. To effectively manage allergies and reduce repeated infections, it is necessary to grasp how these immune mechanisms can malfunction. Such immune irregularities may explain why some children face recurring infections or experience worsening allergic symptoms.

An examination of innate immunity reveals that interferons (IFNs) serve a multifaceted role in patient defense systems. While the body produces IFNs to fight viral invaders, it has been observed that this protective mechanism can sometimes spiral into an excessive immune reaction when infections recur [13]. Studies indicate that higher levels of IFN-γ might actually shield patients from AR, hinting that people with allergies might not produce enough of this crucial cytokine. An important experimental study explored the interplay between cytokines, IFNs, and the immunoregulatory molecule PD-L1 in 80 AR patients, including 18-year-olds. The researchers discovered significantly higher PD-L1 levels in the bloodstream of healthy individuals compared to those with AR, with a clear positive relationship between PD-L1 and IFN-γ concentrations across varying disease intensities [11].

Decreased IFN-γ production has also been observed in disease reinfection. A study involving 72 children compared the innate immune response and pattern recognition receptor expression in allergic asthmatic children. Although IFN-β, IFN-γ, and IL-29 in sputum and plasma were similar between infected and non-infected patients, children with reinfection (rhinovirus and influenza) showed lower levels of IFN-γ in plasma and sputum during exacerbation [20].

Contrary to previous studies, research from the European PreDicta project, a prospective multicenter cohort study, found that when asthmatic children experienced exacerbations, their immune cells produced more IFN-α in response to rhinovirus (RV) infection compared to children without asthma. This could suggest an exaggerated immune response in asthma during infections, potentially related to increased symptom severity [13].

During the SARS-CoV-2 pandemic, lower IFN-α production and an inverse relationship between allergic sensitization and the expression of the angiotensin-converting enzyme 2 (ACE2) receptor, necessary for viral entry into cells, were observed. In individuals with allergic sensitization, lower ACE2 expression was noted, potentially indicating reduced susceptibility to SARS-CoV-2 infection [21]. Another cause of possible protection against SARS-CoV-2, specifically in asthma, may be due to the inhibition of viral replication and relapse of cytokines produced by inhaled corticosteroids and long-acting beta 2-agonists [22]. This suggests that allergic asthma and adequate disease management treatment may act as a possible protective mechanism against SARS-CoV-2 infection. However, there is evidence that associates asthma, specifically severe asthma, with an increased risk of COVID-19 death. A British study analyzing data from a large series of 17 million adults found a 13% increased hazard ratio (CI: 1.01–1.26) for COVID-19 death in patients with severe asthma with recent use of oral corticosteroids. However, the study did not include pediatric patients [23].

Additionally, in the pandemic context, another study observed that bronchial epithelial cells in children with allergic asthma replicated to a lesser extent compared to healthy children, and treatment with IL-13 reduced viral replication in the allergic asthma group. This could be due to changes in the function of ciliated epithelial cells caused by type 2 inflammation, driven by T helper 2 (Th2) lymphocytes, rather than a reduction in ACE2 quantity, which would be more associated with a humoral adaptive response [24]. This protective Th2 response was also observed in another study that found a correlation of 0.89 between eosinophil levels and IgE, produced in response to allergies, in the presence of RV in asthmatic children [25].

Increased IgE production, associated with humoral immunity, has been observed in asthmatic children in response to other viruses. Similar findings of higher IgE production were noted against RV and enterovirus (EV), creating an immune environment conducive to overcoming infection but potentially exacerbating asthma symptoms [14,26].

In relation to adaptive immunity, asthma appears to reduce immune capacity, specifically cell-mediated immunity (CMI), during rhinovirus (RV) infections. This reduction may be due to a decrease in regulatory T cells, potentially leading to difficulties in adequately controlling the immune response to RV infections [6,27].

A cohort study investigating biomarkers in nasal washes, lung function, and viral infection in asthmatic children over a year found that immune system changes in the presence of allergic diseases like asthma affect the response to viral infections. This includes airflow obstruction influenced by specific pro-inflammatory cytokines and a less severe reduction in certain adaptive immunity biomarkers in the presence of viruses [4].

Comparing nasal cytokine responses to natural RV infections in healthy and asthmatic children, we identified higher basal expression of IL-8, IL-12, and IL-1β in asthmatic children. When infected with RV, both groups experienced increased interferon production, but asthmatic children also showed increased IL-8, IL-13, and IFN-β, affecting both innate and adaptive immunity [5]. The type of pathogen can influence immune response capacity; for example, RV-C induces lower IFN-γ production and does not increase Th2 cell activity compared to RV type A [28].

Investigating the effect of RV on bronchial epithelial tight junctions found that RV infection causes a more sustained alteration in airway barrier function in asthmatic children compared to non-asthmatic ones, suggesting greater susceptibility and severity to respiratory infections [29]. Additionally, RV infections could alter DNA methylation and mRNA expression in asthmatic children, negatively affecting immune response and airway inflammation, contributing to asthma development and severity [30].

In the case of bacteria that can cause respiratory infections, allergic asthma has shown potential protective capacity in CMI against Chlamydia pneumoniae (Cp), evidenced by higher IFN-γ production compared to non-asthmatic children [31]. However, another study on Mp in asthmatic children found alterations in CMI but did not conclusively determine if asthma played a protective role. Observations included a decrease in CD3+ and CD4+ T lymphocytes but an increase in CD8+ T lymphocytes [32].

In short, alterations in the immune response, both innate and adaptive, of children with allergies such as asthma may significantly could influence their susceptibility to recurrent infections. These changes range from alterations in interferon production to variations in cytokine expression and immune cell function.

## 5. Infections Associated with Allergic Diseases

The interaction between allergic diseases and infections is complex and varied. The presence of allergies, such as asthma and RA, is often associated with an increased susceptibility to certain infections that may even aggravate the underlying allergic disease or illness. This makes it important to understand the links between allergic diseases and different types of infection in order to improve treatment and intervention strategies in pediatric patients.

### 5.1. Viral Infections

Viral respiratory infections are especially common in children with allergies and asthma, with RV being one of the most common agents in patients with allergic asthma [14,27]. A prospective study of 1089 children from birth to 2 years old revealed that 12% of those with recurrent respiratory infections were diagnosed with asthma. In this group, RV was identified in 58% of cases, and 60% experienced at least three episodes of acute otitis media. These findings suggest that recurrent respiratory infections in early childhood, especially those caused by RV, may also be a risk factor for the development of asthma [15].

The relationship between SARS-CoV-2 and allergic diseases has also been studied, possibly having no concrete impact on disease progression. A retrospective analysis of 46,900 children treated at the Duke University Health System found no significant differences in the risk of SARS-CoV-2 infection between children with and without asthma [33]. At another pediatric center, 38 children hospitalized with COVID-19 were analyzed, and it was observed that 34.2% had asthma. These asthmatic children did not show a more severe course of the disease and required fewer medical interventions [34]. Additionally, a cross-sectional study identified a prevalence of 5.9% of AR among 322 patients with COVID-19, suggesting that AR may not influence the severity of the disease [7]. An additional analysis of 75 pediatric patients with COVID-19 and allergies showed that those with AR or allergen sensitization presented milder cases of COVID-19 [35].

Concerning viruses affecting the gastrointestinal and respiratory systems, the scientific literature is inconclusive regarding the association between EV and allergies. One study found no significant differences in the frequency of EV infections between children with and without allergic sensitization, although children with allergic diseases, especially atopic dermatitis, presented fewer EV infections [36]. On the other hand, another cohort study suggests a possible link indicating that EV infection could be related to a higher risk of developing asthma in children, particularly those under 5 years old, with a 1.48 times greater risk [37].

Herpes zoster (HZ) skin infection has also been analyzed in the presence of asthma, showing a possible association. A population-based case–control study observed that children with asthma were more likely to develop HZ, with a 2.56 times greater risk [38]. Likewise, an analysis based on the Taiwan health insurance database found a higher risk of HZ in children with asthma, although regular treatment with inhaled corticosteroids and montelukast was associated with a decreased risk [39].

### 5.2. Bacterial Infections

The presence of bacteria associated with respiratory diseases is common in individuals with asthma. Mp is frequently found in patients with this disease, potentially predisposing them to other respiratory conditions. Atopic children demonstrate significantly higher rates of MP-related pneumonia (27.1%) compared to non-atopic children (4.9%), though this pattern does not extend to AR and atopic dermatitis cases [17]. Among children experiencing acute asthma exacerbations, nearly half (46.15%) test positive for MP-specific IgM antibodies [16]. On the other hand, an association has been observed between invasive pneumococcal disease caused by Streptococcus pneumoniae and asthma, with a threefold increased risk in patients with asthma [18]. Regarding Cp, a case–control study investigated the immune response in children with and without asthma, finding that asthmatic children have a stronger immune response to this pathogen even without the presence of infection, evidenced by their higher levels of IFN-γ [31].

Infection with Helicobacter pylori (H. pylori) could have an inverse relationship with the presence of pediatric asthma. A cross-sectional study of 2241 children found that the asthma diagnosis rate in those with H. pylori positive (3.77%) was significantly lower than in those with H. pylori negative (7.23%) [40]. Another analysis found no statistically significant association between early H. pylori infection and asthma development, although it observed a trend of lower asthma prevalence in children with H. pylori positive at 2 years old, where none of the children with H. pylori positive at 2 years developed asthma at 16 years old. On the other hand, 16.4% of children with H. pylori negative at 2 and 10 years had asthma at 16 years [41]. Additionally, a case–control study also found an inverse association between H. pylori infection and pediatric asthma, with H. pylori IgG seropositivity being lower in asthma cases (25%) than in controls (40%) [42].

Another important bacterial factor in allergic airway diseases is colonization by Staphylococcus aureus, particularly strains that produce enterotoxins. S. aureus enterotoxins function as superantigens by cross-linking MHC class II molecules on antigen-presenting cells with T cell receptors, driving massive, non-specific T cell activation and a surge in Th2 cytokines (IL-4, IL-5, IL-13). This hyper-activated Th2 milieu promotes local IgE synthesis and eosinophilic inflammation, while enterotoxins can also bind TLR2 on epithelial cells to induce IL-6 and IL-8 release, disrupting epithelial barrier integrity and perpetuating type 2 airway inflammation. Sensitization to these enterotoxins has been associated with more severe asthma and chronic rhinosinusitis phenotypes, suggesting that S. aureus-driven superantigen activity represents a promising target for future therapeutic strategies [43,44].

### 5.3. Fungal Infections

Regarding fungal infections, a retrospective study of 150 children hospitalized with asthma identified Candida albicans as the most common fungus (61%) in cases of fungal lung infection. Furthermore, this study found that independent risk factors for developing fungal lung infection included ages under 3 years (OR = 4.865), comorbidities such as rhinosinusitis or AR (OR = 3.241), and more than three asthma attacks during hospitalization (OR = 2.255). The infection was associated with symptoms such as cough, persistent fever, wheezing, dyspnea, crackles, and hepatosplenomegaly [45].

Understanding the relationship between possible recurrent infections and allergic diseases could help in creating proactive and specific management strategies in the pediatric field. Thus, early and specific interventions can be implemented in allergic patients, reducing the disease burden and anticipating the appearance of possible associated diseases.

## 6. Exacerbations of Diseases in Allergy

The presence of Mp and Cp in children with asthma shows higher levels of IgM and IgG in more severe cases of the disease, suggesting an asthmatic exacerbation in the presence of these bacteria [46]. Additionally, Mp has been found to negatively affect T lymphocyte function in bronchoalveolar lavage fluid in asthmatic children during both acute and stable periods, with a decrease in CD3+ and CD4+ T cell counts and an increase in CD8+ T cells. This could be associated with a possible exacerbated inflammatory response, thus aggravating the symptoms of pediatric asthma [32].

Evidence regarding the relationship between influenza and the exacerbation of allergic asthma is not fully defined. For example, research in children hospitalized for respiratory tract diseases determined that having influenza predisposed them to a higher likelihood of asthma exacerbations compared to bronchiolitis or pneumonia [47]. However, another study on pediatric patients with acute respiratory illness found that asthma was not a significant risk factor for developing severe influenza (aOR: 1.35, CI: 0.77–2.35), with severe influenza defined as requiring hospitalization, emergency department visits, and/or a diagnosis of pneumonia within 30 days of symptom onset [48].

In pediatric atopy, research has shown that respiratory syncytial virus (RSV) infection is more common compared to non-atopic children (43.3% vs. 22.8%) [49]. Additionally, children hospitalized for RSV in the first two years of life have a 3.3 times higher risk of asthma hospitalizations compared to children not hospitalized for RSV (RR = 3.3, CI: 3.1–3.5) [50].

In asthmatic children, the detection of human rhinovirus (HRV) in nasal samples is significantly associated with higher probabilities of symptoms such as cough and phlegm (OR = 2.0, CI 95%: 1.4–2.86), wheezing, and chest tightness (OR = 2.34, CI 95%: 1.55–3.52) [51]. The prevalence of HRV in children with acute lower respiratory tract infection shows that those with asthma exacerbation are more likely to be infected with HRV in general, and specifically with the HRV-C subtype, compared to children without asthma [52]. Although the virus genotype alone does not determine the severity of the exacerbation, children under 5 years old, especially boys, infected with HRV-C seem to have up to 3.7 times higher probability of experiencing moderate/severe exacerbations (CI 95%: 1.2–13.4). On the other hand, pollen sensitization during pollen season is associated with a lower likelihood of moderate/severe exacerbations in children infected with HRV-C (*p* = 0.01) [53]. A possible cause of asthma exacerbation could be the higher production of IFN-α in asthmatic children in response to HRV [13].

An analysis of the DOORWAY study, which included 958 children with moderate to severe asthma exacerbations, revealed that the presence of any respiratory pathogen was not associated with greater initial severity but was associated with a higher risk of treatment failure (20.7% vs. 12.5%) [54]. Another prospective study on children with asthma exacerbations found that 81.7% had viruses or atypical bacteria, with RV being the most common (75.1%). Although there was no relationship between the presence of microorganisms and the acute severity of asthma, there was a significant association with relapses and the need for medical attention within the first 14 days (OR = 1.11, CI 95%: 1.00–1.23) [8].

During the SARS-CoV-2 pandemic, asthmatic patients with COVID-19 required fewer medical interventions [34]. However, the virus variant could influence the frequency of exacerbations. A retrospective study of 573 children with asthma found that the proportion of children with COVID-19 experiencing an asthma exacerbation was significantly higher during the Omicron wave (40.2%) compared to the Pre-Delta (22.6%) and Delta (26.2%) waves. The odds of an asthma exacerbation were 2.8 times higher during the Omicron wave than in previous waves (CI: 1.70–4.61) [55]. Furthermore, another study on allergen sensitization and AR found a possible association with the severity of COVID-19. Among asymptomatic patients, 80.8% had mild symptoms and showed significantly higher total IgE levels (median 71.8 IU/mL) compared to those with moderate to critical disease (median 39.1 IU/mL), which could imply a lower exacerbation of the disease in allergic patients [35].

In a retrospective study with 122 patients, the clinical characteristics of asthma sensitized to Aspergillus fumigatus (A.f) and allergic bronchopulmonary aspergillosis (ABPA) were compared. ABPA patients showed higher levels of fractional exhaled nitric oxide (FeNO), eosinophils, specific IgE for A.f, as well as a higher ratio of specific IgE for A.f to total IgE compared to those with A.f-sensitized asthma. FeNO and eosinophils were identified as risk factors for the development of ABPA in patients with A.f-sensitized asthma. Although these results do not determine if this type of infection is more prone in allergic patients, higher levels of FeNO and eosinophilic inflammation could negatively affect the airways, mainly in the context of asthma or AR [56].

## 7. Limitations

It is important to acknowledge the inherent limitations of this study. Although a structured literature search was conducted, this review is narrative in nature, which may limit objectivity and reproducibility compared to systematic reviews. No formal quality assessment or risk of bias evaluation was performed, as this is not standard for narrative reviews. Additionally, the selection and interpretation of studies may be influenced by the authors’ perspectives, and it is possible that some relevant works may not have been identified due to the inherent limitations of the search strategies employed.

## 8. Final Considerations and Future Directions

The evidence presented in this review shows the complex interplay between allergic inflammation and recurrent infections in pediatric patients. While multiple studies have identified immune dysregulation, particularly involving interferons, Th2 cytokines, and epithelial barrier integrity, as a potential mechanism contributing to increased infection susceptibility, a comprehensive understanding of how specific immune pathways interact in allergic children remains incomplete. Notably, gaps persist in delineating the causal directionality of these relationships, identifying reliable biomarkers to predict risk, and differentiating the immune profiles of allergic versus non-allergic children with recurrent infections. Future research should aim to define these immunological signatures with greater precision, ideally through prospective pediatric cohort studies and mechanistic investigations. Clinically, a deeper understanding of these interactions could facilitate earlier identification of high-risk patients and support the development of targeted immunomodulatory therapies aimed at reducing infection frequency and mitigating allergic disease progression.

Beyond the scope of this review, an important clinical factor requiring additional consideration is the role of excessive systemic corticosteroid prescription in allergic pediatric patients. Prolonged oral corticosteroid use, even at moderate doses, can induce secondary immunodeficiency characterized by significant CD4+ lymphopenia and decreased IgG levels, thereby increasing susceptibility to opportunistic and bacterial infections. This practice may create a detrimental cycle where treatment intended to control allergic exacerbations inadvertently compromises immune defenses, predisposing patients to recurrent infections that can subsequently trigger new allergic crises. Therefore, judicious steroid management strategies, including dose and duration limitation, steroid-sparing therapies, and alternate-day regimens, are crucial to minimize immunosuppressive impact while maintaining allergic disease control [57].

## Data Availability

Data sharing not applicable to this article as no datasets were generated or analyzed during the current study.
